# Aging restricts the initial neural patterning potential of developing neural stem and progenitor cells in the adult brain

**DOI:** 10.3389/fnagi.2024.1498308

**Published:** 2025-01-23

**Authors:** Saeideh Aran, Mohammad Ghasem Golmohammadi, Mohsen Sagha, Kamran Ghaedi

**Affiliations:** ^1^Department of Plant and Animal Biology, Faculty of Biological Science and Technology, University of Isfahan, Isfahan, Iran; ^2^Research Laboratory for Embryology and Stem Cells, Department of Anatomical Sciences, School of Medicine, Ardabil University of Medical Sciences, Ardabil, Iran; ^3^Department of Cell and Molecular Biology and Microbiology, Faculty of Biological Science and Technology, University of Isfahan, Isfahan, Iran

**Keywords:** adult brain, aging, ganglionic eminence, neural patterning, neural stem and progenitor cell, neurosphere, subventricular zone

## Abstract

**Introduction:**

Neurosphere culture is widely used to expand neural stem and progenitor cells (NSPCs) of the nervous system. Understanding the identity of NSPCs, such as the principals involved in spatiotemporal patterning, will improve our chances of using NSPCs for neurodevelopmental and brain repair studies with the ability to direct NSPCs toward distinct fates. Some reports indicate that aging can affect the nature of NSPCs over time. Therefore, in this study, we aimed to investigate how the initial neural patterning of developing NSPCs changes over time.

**Methods:**

In this research, evidence of changing neural patterning potential in the nervous system over time was presented. Thus, the embryonic and adult-derived NSPCs for cardinal characteristics were analyzed, and then, the expression of candidate genes related to neural patterning using real-time quantitative reverse transcription polymerase chain reaction (RT-qPCR) was evaluated at various stages of embryonic (E14 and E18), neonatal, and adult brains. Finally, it was assessed the effect of cell attachment and passage on the initial neural patterning of NSPCs.

**Results:**

The analysis of gene expression revealed that although temporal patterning is maintained *in vitro*, it shows a decrease over time. Embryonic NSPCs exhibited the highest potential for retaining regional identity than neonatal and adult NSPCs. Additionally, it was found that culture conditions, such as cell passaging and attachment status, could affect the initial neural patterning potential, resulting in a decrease over time.

**Conclusion:**

Our study demonstrates that patterning potential decreases over time and aging imposes restrictions on preliminary neural patterning. These results emphasize the significance of patterning in the nervous system and the close relationship between patterning and fate determination, raising questions about the application of aged NSPCs in the treatment of neurodegenerative diseases.

## Graphical abstract

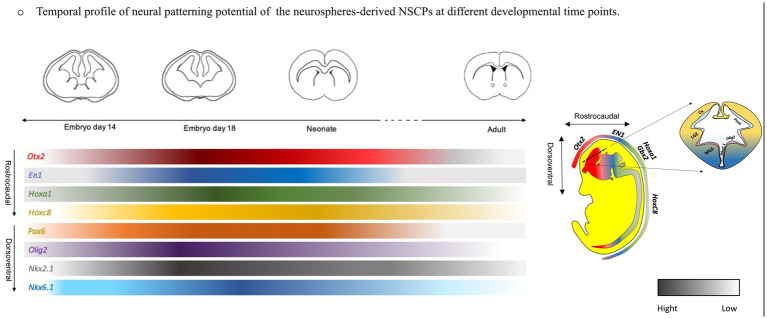

## 1 Introduction

Neurogenesis is a complex and orchestrated series of processes that generate neural networks by organizing neuronal diversity at the appropriate place and time during development. This phenomenon is one of the fascinating events, primarily occurring in the embryonic and neonatal stages, extending to adulthood as well (Muñoz-Sanjuán and Brivanlou, 2002). In the early embryo, an ectodermal sheet is initially organized into spatially confined domains of gene expression with neurogenic potential, leading to the formation of the neural plate, which contains neural progenitor cells with distinct molecular profiles. Subsequently, this neural plate rolls up to form a neural tube that patterns along a rostrocaudal (RC) axis, creating distinct parts of the central nervous system (CNS) including the forebrain, midbrain, hindbrain, and spinal cord. Concurrently, each of these regions is subdivided along the dorsoventral (DV) axis, forming specific domains (Kelly et al., 2009). In the forebrain, the dorsal part of the developing neural tube expands to form the pallium, generating the cerebral cortex, while the ventral part creates the sub-pallium area, giving rise to lateral, medial, and caudal ganglionic eminences (LGE, MGE, and CGE), respectively (Duan et al., 2013). It has been found that various populations of embryonic neural stem cells (NSCs) are localized in a specific area of the telencephalon, including the GE (Llorente et al., 2022). However, in the adult brain, they become more restricted to the dentate gyrus (DG) of the hippocampus (Altman, 1962) and the subventricular zone (SVZ) of the lateral ventricles (Reynolds and Weiss, 1992). These neurogenic niches are specialized microenvironments whose primary purpose is to maintain homeostasis and regulate CNS behavior (Gómez-Gaviro et al., 2012; Riquelme et al., 2008).

Neural stem cells are undifferentiated, self-renewing, and multipotent cells that undergo a variety of molecular and morphological changes and generate various neurons and glial cells, spatiotemporally (Kohwi and Doe, 2013). The morphological and functional properties of NSCs closely align with their molecular features, which are identified by spatiotemporal patterning (Miranda-Negrón and García-Arrarás, 2022). However, during development, intrinsic and extrinsic temporal cues limit the competence of NSCs, leading to the diversity among them from which the specific cell types emerge. This diversity arises from spatial patterning, where various neural progenitor pools are positioned precisely within specific domains of the developing neural tube along the RC and DV axes. These defined domains are characterized by the combinatorial expression of distinct transcription factors (TFs) influenced by different morphogens (BMP, Shh, FGF, and RA) secreted from various regions of the adjacent tissues resulting in the generation of the individual cell population (Beccari et al., 2013; Kiecker and Lumsden, 2012).

Over the last decades, unfolding the development processes, diversity of cell types involved in CNS formation, and the intrinsic and extrinsic modifications that take place in NSCs and their niches with aging has been a major challenge of developmental neurobiology (Kohwi and Doe, 2013). Aging has been shown to have a significant impact on brain function, leading to debilitating effects (Navarro Negredo et al., 2020). These effects may be linked to a decline in age-related cognitive function, which limits CNS plasticity and repair (Encinas et al., 2011). The ability of NSCs to proliferate and generate new neurons diminishes over time (Bailey et al., 2004; Ben Abdallah et al., 2010), adversely affecting the niches and disrupting natural homeostatic functions (Kuhn et al., 1996), ultimately impacting regenerative capabilities (Baker and Petersen, 2018). Age is a significant risk factor for neurodegenerative diseases. Consequently, research on the aging of NSCs has increased, leading to significant advances in understanding the changes these cell populations experience during aging through various *in vitro* and *in vivo* model systems (Nicaise et al., 2020).

Neuronal disorders have various causes, such as deficiencies in NSC propagation and differentiation (e.g., macrocephaly, microcephaly, schizophrenia, autism, and Huntington’s disease), dysfunction of NSCs through inappropriate neuronal transmission (e.g., schizophrenia, epilepsy, autism, and bipolar disorder), and neuronal death (i.e., neurodegenerative disorders) (Liszewska and Jaworski, 2018). These problems led scientists to investigate the potential of NSCs in cell-based therapy as a promising treatment strategy for disorders (Li D. et al., 2022), such as epilepsy (Upadhya et al., 2019), schizophrenia (Gilani et al., 2014), autism spectrum disorder (Southwell et al., 2020), and Alzheimer’s disease (Lu et al., 2020).

Given the complexity and dynamic nature of the nervous system, studying all these activities *in vivo* is a significant undertaking. However, it can be replicated *in vitro* through the development of assays and procedures that mitigate some of the associated complications and enhance the understanding of neurogenesis and related disorders (Azari and Reynolds, 2016). While our knowledge is in its infancy, the growing information on the regulation of competence of NSCs and temporal identity would help increase the efficiency of their direct differentiation *in vitro* toward the specific neural subtype (Rossi et al., 2017) for cell therapy purposes. Thus, in the field of aging studies, age-related stem cell damage is essential for finding new therapies for neuronal disorders. Several studies have shown the age effect on self-renewal of NSCs and neurogenesis capabilities, both *in vivo* and *in vitro* (Encinas et al., 2011), and while signaling molecules can impact neurogenesis in stem cells throughout adulthood, their response to these factors changes with age. It is unclear whether stem cells undergo developmental changes in adults that differ from embryos and are both temporally and regionally limited (Temple, 2001a,b). It is of interest to request whether neural stem cells’ own intrinsic regional identity, for example, can be conserved in the lack of their *in vivo* condition (Hitoshi et al., 2002), or whether NSCs in the developing brain generally lack regional identity or positional information which changes over time (Temple, 2001a,b).

The previous study suggested that NSC colonies isolated from the spinal cord and cortex of E14.5 mice express regional genes differently along the RC axis (Zappone et al., 2000). However, much remains unclear regarding the potential of neural patterning in NSPCs and alterations in inherited patterning over time, both within the brain and *in vitro* culture condition. Herein, we aimed to investigate the influence of the temporal patterning on the gene expression profile of NSPCs from embryonic, neonate, and adult mouse brains, providing detailed information on the similarities and differences between properties of embryonic and adult NSPCs to facilitate the manipulation of NSPCs in the future. For this purpose, the neurosphere assay was used to expand the NSPCs population. We found that NSPC-derived neurospheres generated from embryonic GE, neonate, and adult SVZ exhibited different gene patterning profiles, suggesting that the ability to maintain the initial neural patterning diminishes over time.

## 2 Materials and methods

Dulbecco’s Modified Eagle Medium (DMEM)/F12, HEPES buffered saline solution, fetal bovine solution (FBS), and trypsin–EDTA solution were purchased from Biowest (France, Europe). Epidermal growth factor (EGF) and basic fibroblast growth factor (bFGF) were obtained from STEMCELL Technologies (Vancouver, Canada). Penicillin and Streptomycin 100x (Pen/Strep) was purchased from Biotech (Stadtallendrof, Germany). Trypsin inhibitors and heparin were provided by Sigma-Aldrich (St. Louis, MO, United States); 100x GlutaMAX, non-essential amino acid (MEM NEAA), B27 supplement 50x, N2 supplement 100x, and fetal calf serum (FCS) were purchased from Gibco (Grand Island, USA). TRIzol and albumin were purchased from Biobasic (Markham, Canada) and DMSO from Denazist (Mashhad, Iran). Ethanol, chloroform, and isopropanol were provided by Merck (Darmstadt, Germany). cDNA Synthesis Kit and Real Q Plus 2x Master Mix Green were purchased from Sinaclon (Tehran, Iran).

### 2.1 Animals

In this experimental study, 25 adult male and 25 adult female BALB/c mice weighing approximately 25–30 g were purchased from the animal house of Royan Institute, Tehran, Iran. The animals were kept in standard polypropylene cages (four mice per cage) with well-aerated stainless steel frames and had free availability to water and chow pellets. The strict and controlled conditions for the maintenance of the mice were as follows: a temperature of 22 ± 2°C, a humidity of 40–45%, and 12-h/12-h light–dark cycles.

### 2.2 Isolation and cultivation of NSPCs from adult mouse brain

Eight-week-old male mice (*n* = 25) were killed by cervical dislocation, and brains were isolated from the skull using a small spatula following rinsing with 70% ethanol for sterilization. The brains were sectioned coronally at the optic chiasm and olfactory bulb. Then, a thin layer of SVZ, approximately 1.5 × 0.5 mm^2^ of the lateral wall of the ventricles toward the rostral part of the mouse brain, was isolated with a pair of fine curved forceps and then digested enzymatically with 0.05% trypsin–EDTA solution. The isolated cells were primarily seeded in T25 flasks at a concentration of 5×10^4^ cells.ml^−1^ using prepared NSC basal medium containing DMEM/F12 complemented with 2% B27, 1x GlutaMAX, 1% NEAA, heparin (1 μL.ml^−1^ of 0.2%), 100 U/mL penicillin and 100 μg.ml^−1^streptomycin, EGF (20 ng.ml^−1^), and bFGF (10 ng.ml^−1^). After 6–7 days of incubation in a humidified incubator with 5% CO_2_ at 37°C, SVZ-derived neurospheres with an average diameter of 150–200 μm were obtained and used for further passages (Azari et al., 2010; Walker and Kempermann, 2014).

### 2.3 Isolation and cultivation of NSPCs from postnatal mouse brain

Mice (*n* = 10) were killed via cervical dislocation at 3 to 5 postnatal days (P3-5). Then, the mouse head was rinsed with 70% ethanol for sterilization, and the brain was isolated from the skull using a spatula. Under a dissecting microscope, the SVZ zones were removed using a scalpel blade following coronal slices in an anterior-to-posterior axis and were dissected as in the previous section discussed for primary cell culture. The SVZ single-cell suspension was diluted and seeded at a concentration of 4×10^4^ cells. ml^−1^ in T25 flasks using prepared DMEM/F12 complemented with 2% B27, 1% GlutaMAX, 1% NEAA, heparin (1 μL.ml^−1^of 0.2%,), 100 U/mL penicillin and 100 μg.ml^−1^ streptomycin, EGF (10 ng.ml^−1^), and bFGF (5 ng.ml^−1^). On days 6 to 8 of incubation in an incubator with 95% humidity, 5% CO2, and 37°C, SVZ-derived neurospheres with an average diameter of 150–200 μm were obtained and used for further passages (Soares et al., 2020).

### 2.4 Isolation and cultivation of NSPCs from embryonic mouse brain

Female mice (*n* = 25) on days 14 and 18 of gestation were sacrificed by cervical dislocation and their abdomens were washed with 70% ethanol for sterilization of the area. The skin of the abdomen was caught using forceps and trimmed. Consequently, the embryos were collected into HEPES solution with 10% antibiotics. To remove the brain from the skull, the head was held by curved forceps from the caudal side and the dorsal part was facing upward. Thereafter, a horizontal section was made above the nose and a sagittal section was made from the forehead toward the back of the head using micro scissors. Pushing the edges of the trimmed section, the brains were harvested from the skull. In a dorsal side facing, the ganglionic eminences (GE) were exposed and dissected out following a section across the cortex of every hemisphere from the olfactory bulbs to the back of each hemisphere. For isolating NSPCs, the dissected GE tissues were chopped and gently pipetted in the medium until a homogeneous single-cell suspension was acquired. The cells were put in T25 flasks at a concentration of 2×10^5^ cells.ml^−1^ using a prepared basal medium containing DMEM/F12 complemented with 2% B27, 1% GlutaMAX, 1% NEAA, heparin (1 μL.ml^−1^ of 0.2%,), 100 U.ml^−1^ penicillin and 100 μg.ml^−1^ streptomycin, and EGF (20 ng.ml^−1^). After 3–4 days of incubation in a condition of 95% humidity, 37°C, and 5% CO_2_ primary neurospheres were acquired and used for further passages (Azari et al., 2011a,b).

### 2.5 Differentiation of neural stem cells

The E14 mouse brain-derived neurospheres after 4–5 days from the second passage, when the diameter reached approximately 150 μm (measured by the Olympus CKX41 inverted microscope equipped with DP27 camera connected to Cellsense software, standard 1.14.), were trypsinized with 1 mL of Trypsin–EDTA solution for 2 min at room temperature (RT). Then, trypsin neutralization was performed by adding equal volume of trypsin inhibitor to the suspension. The solution was pipetted and transferred to a centrifuge at 700 rpm (110 g) for 5 min. Then, the pellets were transferred to 1 mL of basal medium containing DMEM/F12 complemented with 2% B27, 1% GlutaMAX, 1% NEAA, heparin (1 μL.ml^−1^ of 0.2%,), 100 U.ml^−1^ penicillin, 100 μg.ml^−1^ streptomycin, and EGF (20 ng.ml^−1^) with 5% fetal bovine serum (FBS). The mixture was pipetted approximately 5 to 7 times until it formed milky cell suspensions, with no remaining neurospheres. The cells were then seeded at a concentration of 5×10^5^ cells.ml^−1^ onto the poly-L-ornithine-coated 24-well culture dishes using 1 mL of the same medium for 3–4 days. Subsequently, the medium was changed to the same formulation containing 5% FBS but lacking any growth factors. After 21 days, the cells extracted from the neurospheres will be adequately differentiated (Azari et al., 2011a,b; Chaddah et al., 2012; Li et al., 2018).

### 2.6 Immunofluorescence staining

The stemness and differentiation characteristics of NSPCs were evaluated by immunofluorescence staining. The neurospheres were washed with PBS and fixed using 4% paraformaldehyde for 15 min at RT. Then, they were set in a blocking buffer containing 10% goat serum and 0.3% Triton X-100 for 30 min at RT. The neural stem cell, neuronal, and glial cell markers were determined using primary antibodies including rabbit anti-*Nestin* (Chemicon, 1:200), mouse anti-*ß-Tubulin III* (G7121, Promega, 1:2000), rabbit anti-*GFAP* (Z0334, DakoCytomation, 1:500), and rat anti-*MBP* (MAB386, Chemicon, 1:200) overnight (Table 1). Donkey anti-rabbit Alexa Fluor 488 (green) (A21206, Invitrogen), goat anti-mouse Alexa Fluor 568 (red), and goat anti-rabbit Alexa Fluor 350 (blue) were the utilized secondary antibodies for staining for 60 min at RT (Table 2). The cell nuclei were counterstained with *DAPI*, and the images were acquired with a fluorescence microscope (TE2000; Nikon Tokyo, Japan) (Miri et al., 2021).

**Table 1:** Primary antibodies.

Name	Company	Concentration
Mouse anti-ß-Tubulin III	Promega	1:2,000
Rabbit anti-GFAP	DakoCytomation	1:500
Rat anti-MBP	Chemicon	1:200
Rabbit anti-Nestin	Chemicon	1:200

**Table 2:** Secondary antibodies.

Name	Company	Concentration
Goat anti-Mouse Alexa Fluor 568	Molecular Probe	1:1,000
Donkey anti-Rat Alexa Fluor 488	Molecular Probe	1:1,000
Goat anti-Rabbit Alexa Fluor 350	Molecular Probe	1:100

### 2.7 RT-qPCR analysis of neural patterning genes

The expression level of candidate neural patterning genes in RC (*Otx2, En1, Hoxa1*, and *Hoxc8*) and DV (*Pax6, Olig2, Nkx2.1*, and *Nkx6.1*) axes were measured by RT-qPCR. Initially, the total RNA content of cultured neurospheres was extracted using a TRIzol Reagent Kit according to the manufacturer’s protocol (Biobasic, Markham, Canada). The quality and concentration of the extracted RNA at 260/280 nm were assessed by spectrophotometer. Complementary DNA (cDNA) was synthesized using a RevertAid First Strand cDNA Synthesis Kit according to the manufacturer’s protocol (Sinaclon, Tehran, Iran) (Supplementary Table 1). RT-qPCRs were carried out on Light Cycler 96 Real-Time PCR system. Sequences of the used primers were presented in Table 3. The amplification program was as follows: A single step of initial denaturation at 95°C for 10 min followed by 40 cycles of denaturation at 95°C for 15 s, annealing at (58–61°C zone) for 30 s, and extension at 72°C for 25 s. A final cycle of melting was also included. The expression of the *GAPDH* gene as an internal control was evaluated as well. The results were obtained using the 2^-ΔΔCt^ method.

**Table 3:** List of sequences of primers for RT-qPCR.

Gene name	Forward sequence	Reverse sequence
*En1*	5’AACACAACCCTGCGATCCTAC3`	5’TAGCTTCCTGGTGCGTGGAC3`
*Pax6*	5’AGTGAATGGGCGGAGTTATG3`	5’GAACTTGGACGGGAACTGAC3`
*Olig2*	5’GCAGCGAGCACCTCAAATC3`	5’ATCATCGGGTTCTGGGGAC3`
*Otx2*	5’GACGACATTTACTAGGGCACAG3’	5’CTCACTTTGTTCTGACCTCCATTC3’
*Hoxa1*	5’CACGCCAGCCACCAAGAAG3’	5’CGTAGCCGTACTCTCCAACTTTC3’
*Hoxc8*	5’CTAACAGTAGCGAAGGACAAG3’	5’TCTCTAGTTCCAAGGTCTGATAC3’
*Nkx6.1*	5’GAAGAGAAAACACACCAGACCCAC3’	5’GAACCAGACCTTGACCTGACTC3’
*Nkx2.1*	5’AGCATCGGAAGGGAAAACTG3’	5’CGTGTGCTTTGGACTCATCG3’
*Nestin*	5’GTCTACAGGCAGCGCTAACA3’	5’TGGGCATCTGTCAAGATCGG3
*B-Tubulin III*	5’TGAGGCCTCCTCTCACAAGT3’	5’GTCGGGCCTGAATAGGTGTC3’
*Map2*	5’ATACCAGCCGTTTGAGA3’	5’TTCATCTTTCCGCTCGTCGG3’
*GAPDH*	5’GAAGAGAAAACACACCAGACCCAC3’	5’AATCTCCACTTTGCCACTGC3’

### 2.8 Statistical analysis

All experiments were statistically analyzed using GraphPad Prism 8 (GraphPad Software, Inc., San Diego, CA), and data were expressed as Mean ± SEM in at least three independent experiments. To assess significant differences (*p < 0.05*) among groups, independent *t*-test, and one-way ANOVA followed by *post-hoc* analysis with Tukey’s test were utilized, with significant values of ^*^p < 0.05, ^**^p < 0.01, ^+^*p* < 0.001, and ^#^*p* < 0.0001.

## 3 Results

### 3.1 Isolation and culture of NSPCs

**Figure 1:**
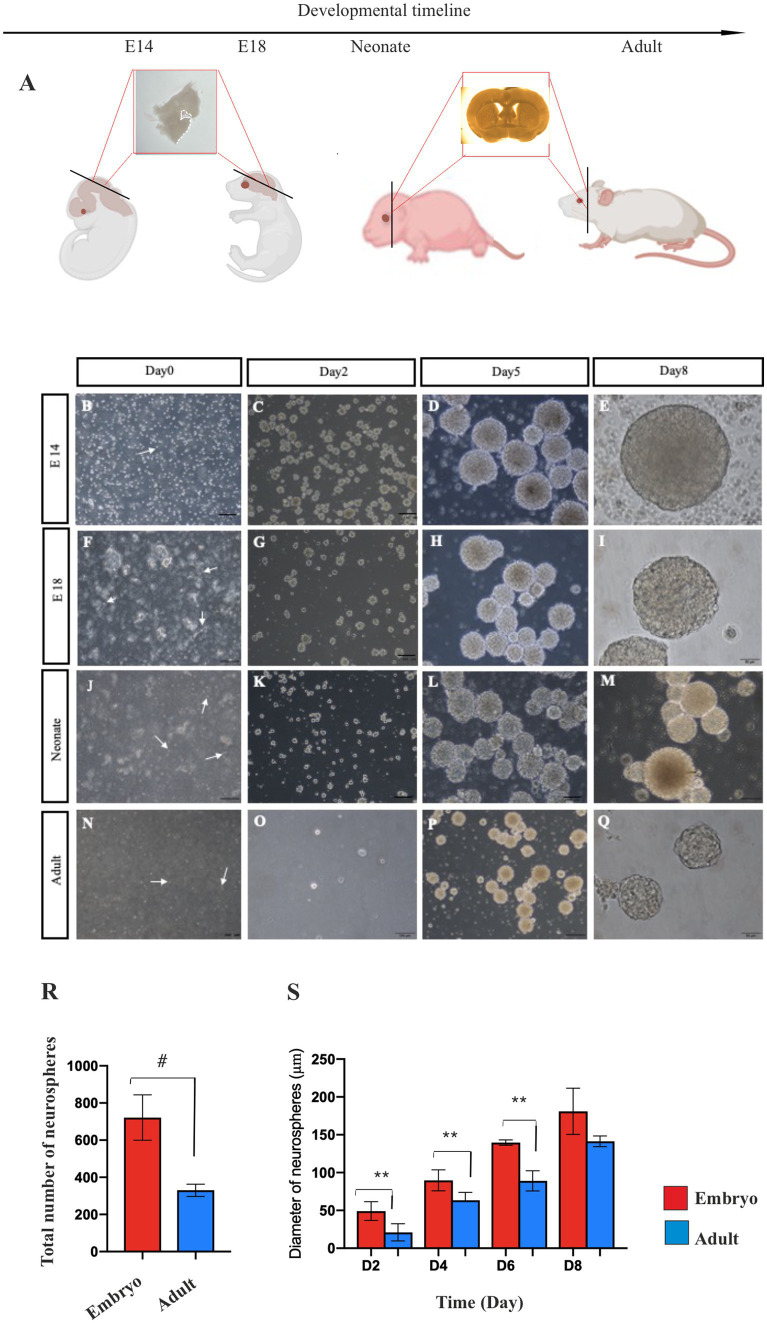
Primary NSPCs culture. Coronal section of mouse brain according to the developmental timeline **(A)**. Primary culture of E14 **(B–E)** and E18 **(F–I)** GE-derived NSPCs, and neonate **(J–M)** and adult **(N–Q)** SVZ-derived NSPCs in different times after isolation. Arrows show single bright NSPCs. Counting of E14 and adult neurospheres per field displays more than two times the number of generated neurospheres in embryonic GE than the adult SVZ **(R)**. E14 GE-derived neurosphere demonstrates a larger size than the adult neurosphere after 2, 4, 6, and 8 days of culture **(S)**. Scale bars represent 100 μm. **p < 0.01, and ^#^*p* < 0.0001.

Cells from the GE of E14 and E18 mouse embryos, the SVZ of the P4 neonate, and the SVZ of 2-month-old adult mice (Figure 1A) were cultivated in a serum-free culture medium supplemented with EGF and bFGF growth factors. Under these conditions, the isolated single cells showed a bright body (Figures 1B,F,J,N) and after 24–48 h of incubation, primary multicellular colonies with a heterogeneous appearance were formed (Figures 1C,G,K,O) which exhibited somewhat homogenous appearance by day 5 (Figures 1D,H,L,P) and reached their optimum size (~150 μm in diameter) with a clear peripheral margin (Figures 1E,I,M,Q) on days 7–8 post-culturing. These neurospheres demonstrated two cardinal properties of NSPCs. They were capable of both self-renewal/expansion and differentiation into multiple neural cell types.

To compare the number of neurospheres derived from embryonic and adult brains in primary culture, manual counting of neurospheres was conducted using an inverted microscope at 4x magnification after isolating and cultivating NSPCs at passage 0 in T25 flasks. This counting was conducted for embryonic neurospheres 5–6 days after culture and adult neurospheres 7–8 days after culture, as the approximate size of neurospheres at these times is approximately 150 μm. Compared to the embryonic brain, the initial density of NSPCs isolated from the adult mouse brain was quite low (Figure 1R). Consequently, as anticipated, only a few numbers of neurospheres derived from the adult brain (298.6 ± 26.03) were generated in primary culture vs. embryonic brain (700.8±17.5) (*p < 0.0001*). In contrast, when comparing the size of neurospheres on days 2–8 of culture, embryonic NSPCs exhibited significantly greater self-renewal/expansion potential than adult NSPCs *(p < 0.01)* (Figure 1S; Supplementary Table 1).

### 3.2 Characterization of NSPCs

**Figure 2:**
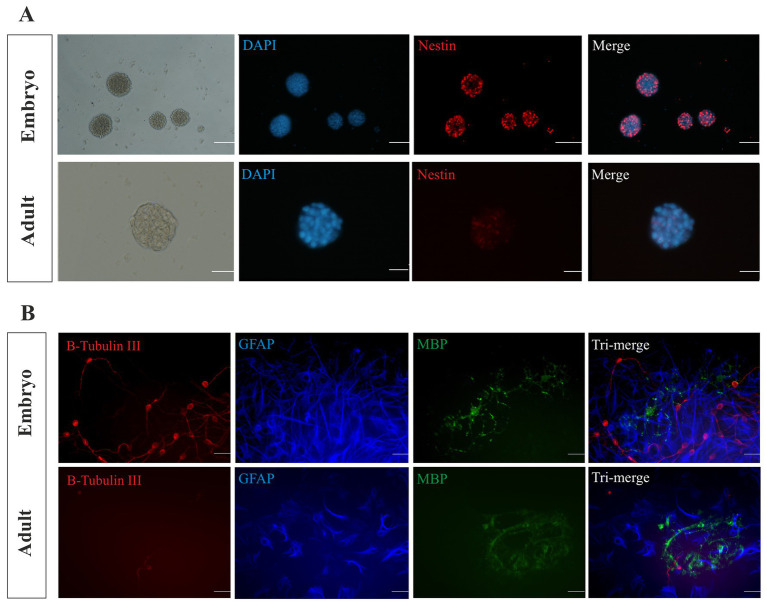
Characterization of NSPCs. The isolated E14 and adult neurospheres were characterized using immunocytochemistry. *Nestin* biomarker analysis confirmed the stemness of the cultured neurospheres **(A)**. Scale bars represent 100 μm for embryonic and 50 μm for adult. Immunocytochemistry analysis displays the differentiation potential of the embryonic and adult NSPCs. *B-Tubulin III-*expressing neurons (red), *GFAP*-expressing astrocytes (blue), and *MBP-*expressing oligodendrocytes (green) were identified and merged **(B)**. Scale bars represent 20 μm.

The immunocytochemistry assay was utilized to characterize E14 and the adult neurospheres, and the expression of *Nestin* as an NSC marker was determined, confirming the neural stemness identity of cultured neurospheres (Figure 2A). We also found that the embryonic and adult NSPCs have the potential to differentiate into neurons, astrocytes, and oligodendrocytes and expressed neural cells biomarker *B-Tubulin III* (*B-Tubulin III,* neuron), glial fibrillary acidic protein (*GFAP,* astrocytes), and myelin basic protein (*MBP,* oligodendrocytes) in dissociated cells after 21 days of incubation in differentiating medium (Figure 2B).

### 3.3 Regional identity characterization of embryonic and adult NSPCs

**Figure 3:**
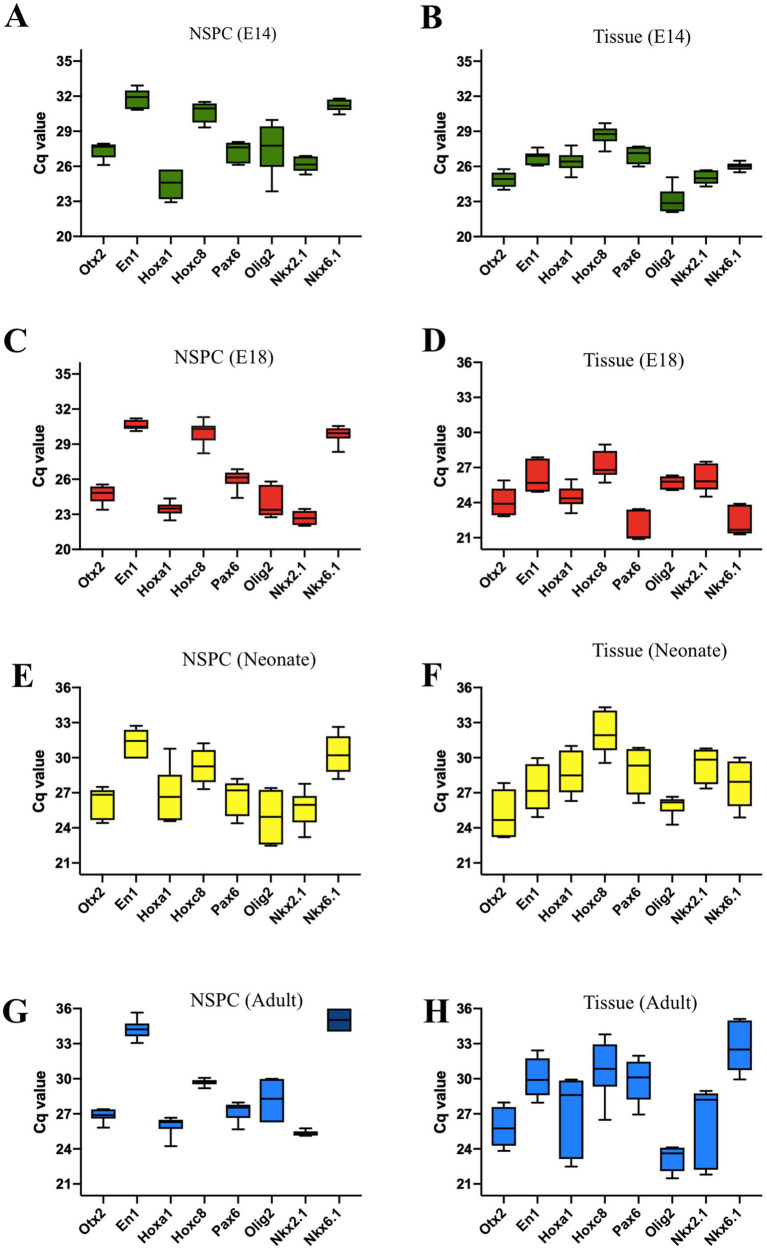
Quantification of regional identity data for E14, E18, neonate, and adult NSPCs and tissues is based on the Cq values of the patterning genes. The characterization of the RC transcription factors reveals the highest expression levels for *Otx2* and *Hoxa1*, with the lowest levels for *En1* and *Hoxc8*. Expression data for GE and SVZ tissues clearly indicate a telencephalic character. The patterning gene expression profile of NSPCs in the DV axis indicates that they are ventrally characterized as belonging to the embryonic GE and adult SVZ regions. These tissues show high expression of *Olig2* and *Nkx2.1*, the GE markers, and low expression of *Pax6* and *Nkx6.1* **(A–H)**. Data are presented as means ± SEM from three independent experiments.

*Otx2, En1, Hoxa1, and Hoxc8* are patterning TFs expressed along the RC axis of the developing neural tube. In the telencephalic area, *Pax6, Olig2, and Nkx2.1* are expressed DV axis, while *Nkx6.1* is expressed in the ventral region of the developing neural tube. The initial neural patterning of GE and SVZ-derived NSPCs was characterized, indicating that both embryonic and adult NSPCs remarkably expressed *Otx2* and *Hoxa1* at higher levels, whereas *En1* and *Hoxc8* were found to be downregulated, demonstrating the rostral identity of isolated NSC in the RC axis. In the DV axis, both embryonic GE and adult SVZ clearly displayed upregulation of *Olig2* and *Nkx2.1* along with lower expression of *Pax6* and *Nkx6.1*, representing the initial ventral patterning of GE and SVZ (Figures 3A–H; Supplementary Table 2).

### 3.4 Potential of neural patterning in embryonic and adult brain

**Figure 4:**
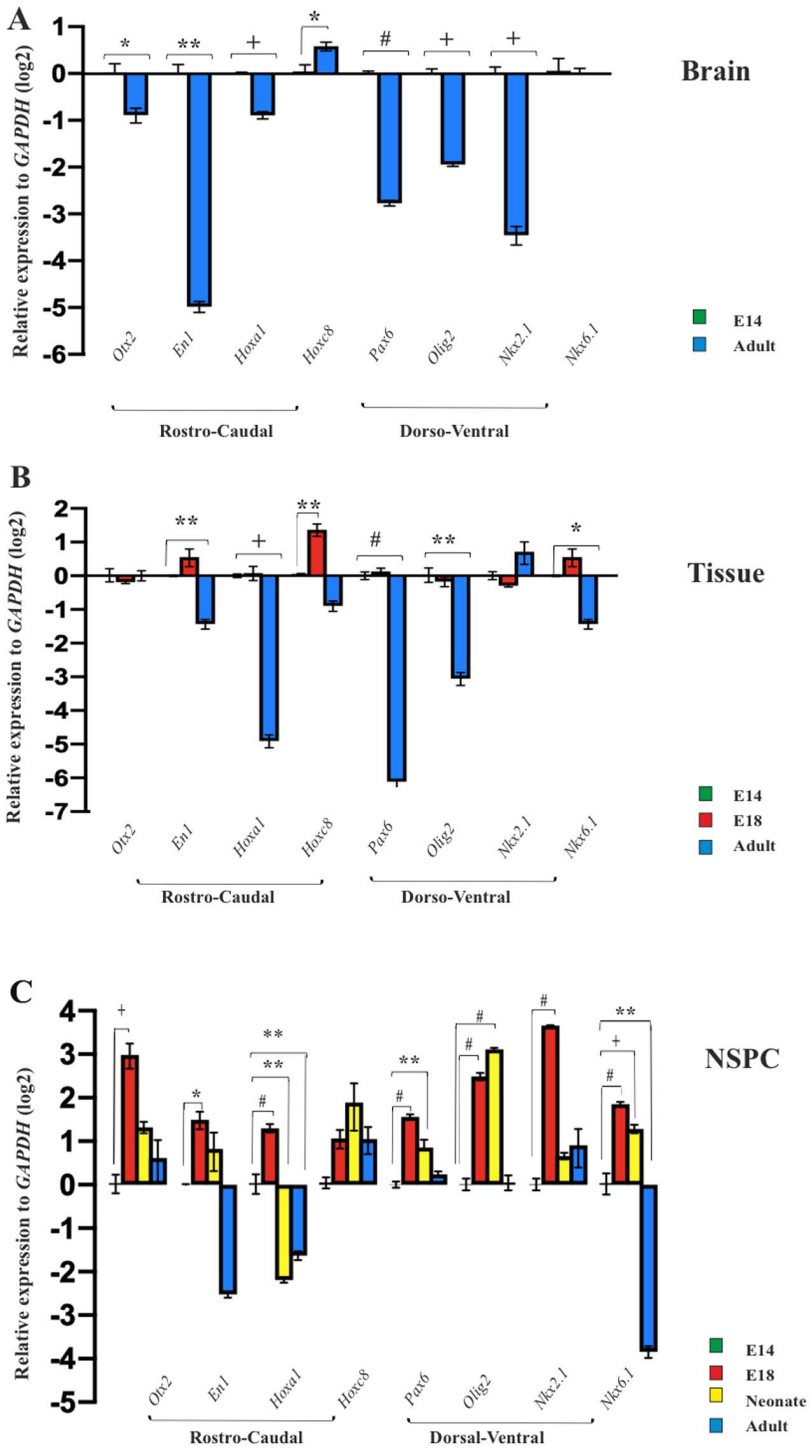
Temporal regional identity of NSPCs and brain. RT-qPCR analysis of the neural patterning genes in embryonic E14 and adult brain **(A)**, E14 GE, E18 GE and adult SVZ **(B)**, and embryonic E14, E18, neonate and adult-derived NSPCs **(C)**. Data are presented as means ± SEM, normalized to the expression level of *GAPDH* as an internal control and compared with the expression level of E14-derived brain, tissue and NSPCs, respectively. ^*^p < 0.05, ^**^p < 0.01, ^+^*p* < 0.001, and ^#^*p* < 0.0001.

The brain is a vital organ that starts its development early in the embryo and continues into the postnatal period (La Manno et al., 2021). To explore whether the long-lasting development of the brain effects on its initial neural patterning, we isolated the embryonic forebrain and the adult brain to display their expression profile of neural patterning genes over time. Compared to the embryonic forebrain, the expression of patterning genes (with the exception of *Hoxc8* expression) was found to be decreased in the adult forebrain, suggesting that the brain alters its initial neural patterning potential by aging (Figure 4A).

### 3.5 Potential of neural patterning in embryonic GE and adult SVZ tissues

Ganglion eminence of the embryonic brain and SVZ in the adult CNS serve as localizing centers for NSPCs (Chen et al., 2017). Consequently, GEs from E14 and E18 along with SVZ from the adult brain, were isolated to investigate the expression profiles of regional identity genes in these tissues at the mRNA level. Our results revealed different levels of gene expression in the GE of the developing brain on days 14 and 18 as well as in the SVZ of the adult brain. The GE on E18 indicated similar or higher levels of patterning gene expression than the E14. However, as expected, the expression levels of the selected patterning genes in SVZ tissues were more restricted and significantly decreased, suggesting that the adult brain fails to maintain the initial expression of neural patterning genes (Figure 4B**)**.

### 3.6 Potential of neural patterning of NSPCs from embryonic into adult

The NSPCs residing in the embryonic GE and adult SVZ are exposed to different niches over time (Li et al., 2024; Navarro Negredo et al., 2020; Nicaise et al., 2020; Solano Fonseca et al., 2016). This raises the question of whether they can retain their initial potential for neural patterning during the prolonged development of CNS (Capela and Temple, 2006). To address this query, NSPCs from E14 and E18 GE, as well as SVZ of neonate and adult brains, were isolated, cultured for neurosphere generation, and passaged one time for ongoing evaluation of RC and DV patterning. Compared to E14 GE-derived NSPCs, cultured neurospheres at passage 1 in the other groups confirmed our previous data and exhibited a conserved region-specific gene expression profile at the cellular level. In particular, the highest expression of patterning genes in E18 GE-derived NSPCs was not retained in neonate and adult SVZ-derived NSPCs, suggesting the decline of the neural patterning potential over time (Figure 4C; Supplementary Table 3).

### 3.7 Evaluation of time-course differentiation status of NSPCs

**Figure 5:**
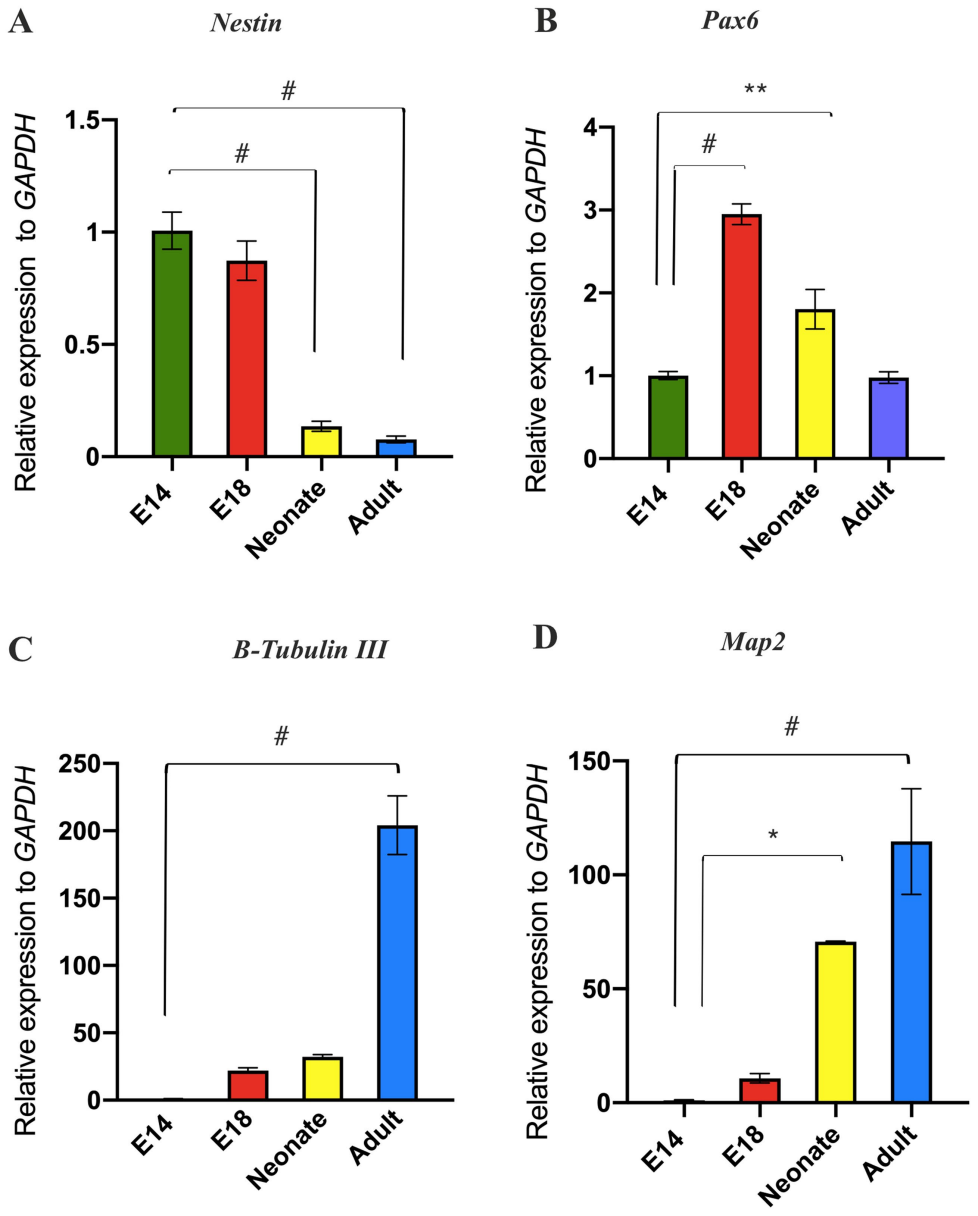
Evaluation of temporal differentiation status of NSPCs. RT-qPCR analysis of stemness markers (*Nestin* and *Pax6*) in **(A)** and **(B)**, respectively and neuronal markers (*B-Tubulin III* and *Map2*) in **(C)** and **(D)**, respectively in E14, E18, neonate, and adult NSPCs. The results indicated that NSPCs lose their stemness properties and differentiate into neural cells over time. Data are presented as means ± SEM, normalized to the expression level of *GAPDH* as an internal control, and compared with the expression level of E14 NSPCs. ^*^p < 0.05, ^**^p < 0.01, ^+^*p* < 0.001, and ^#^*p* < 0.0001.

In the particular domain of the RC and DV axes of the developing neural tube, the profile of the regional-specific genes transitions from the stemness state to a differentiated state (González-Iglesias et al., 2024). This raises the question of whether the decreased expression of neural patterning genes over time may relate to alterations in the differentiation status of NSCs from the embryonic to the adult brain. To address this query, we analyzed stemness-related markers (*Nestin* and *Pax6*) and differentiating neuronal markers (*Map2* and *B-Tubulin III*) in NSPCs. We found that *Nestin* expression diminished in adult SVZ-derived NSPCs compared to E14 GE-derived NSPCs; however, E18 GE-derived NSPCs exhibited a high expression of *Pax6* compared to the other groups, confirming the peak neural patterning potential of the developing brain at this stage (Figures 5A,B). Concurrent with the reduced expression of the neural stemness genes, *B-Tubulin III* and *Map2* were found to be expressed over time, suggesting a more differentiated state in the neonate and adult SVZ than the developing GE (Figures 5C,D; Supplementary Table 4).

### 3.8 Potential of neural patterning in NSPCs vs. tissues of origin

**Figure 6:**
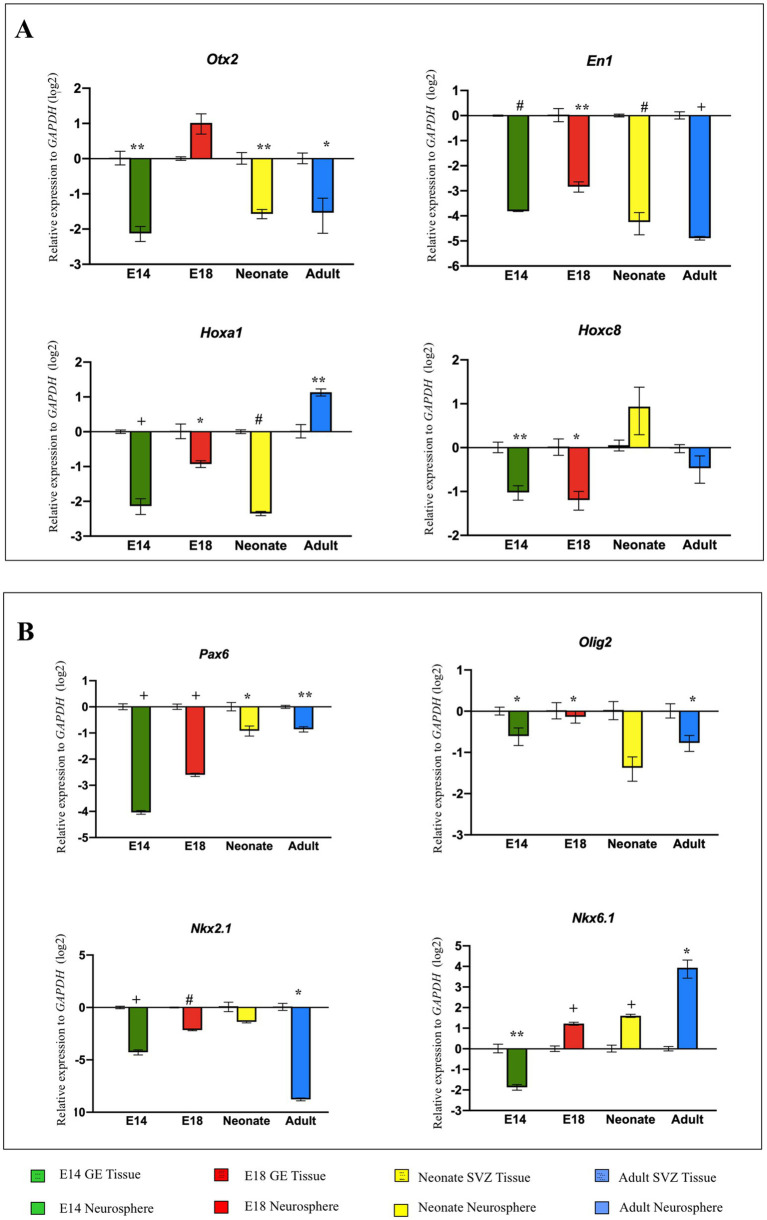
Comparison of neural patterning between NSPCs *vs* tissues of origin. RT-qPCR analysis of gene expression level of RC axis (*Otx2, En1, Hoxa1*, and *Hoxc8*) **(A)** and DV axis (*Pax6, Olig2, Nkx2.1,* and *Nkx6.1*) **(B)** in NSPCs at different developing times become downregulated in comparison with the tissues of origin (GE and SVZ). Data are presented as means ± SEM, normalized to the expression level of *GAPDH* as an internal control, and compared with the expression level of GE of E14, GE of E18, SVZ of neonate, and SVZ of adult in each diagram. ^*^p < 0.05, ^**^p < 0.01, ^+^*p* < 0.001, and ^#^*p* < 0.0001.

Different types of NSCs are populated in both developing GE and SVZ including types A, B, and C, which are highly spatially organized and perform specific functions. Using neurosphere assay, it is impossible to distinguish these NSPC population *in vitro* (Azari and Reynolds, 2016). Therefore, as the pattern formation and regional identity of the developing brain result from its partitioning along the RC and DV axes at the tissue level, we experimentally compared the neural patterning of unknown types of NSPCs with the tissues (i.e., GE/SVZ) from which they originated. Our results indicated that expression of the most patterning TFs, with the exception of *Otx2* (E18), *Hoxa1* (Adult), *Hoxc8* (Neonate), and *Nkx6.1* (Adult), in NSPCs decreased compared to the host tissues, suggesting that NSPCs are not capable of retaining tissue-specific patterning upon isolation. Among the different groups, E18-derived NSPCs demonstrated a considerably better ability to maintain this regional identity potential than NSPCs from other groups (Figures 6A,B; Supplementary Table 5).

### 3.9 Potential of neural patterning in embryonic and adult NSPCs in different passages

**Figure 7:**
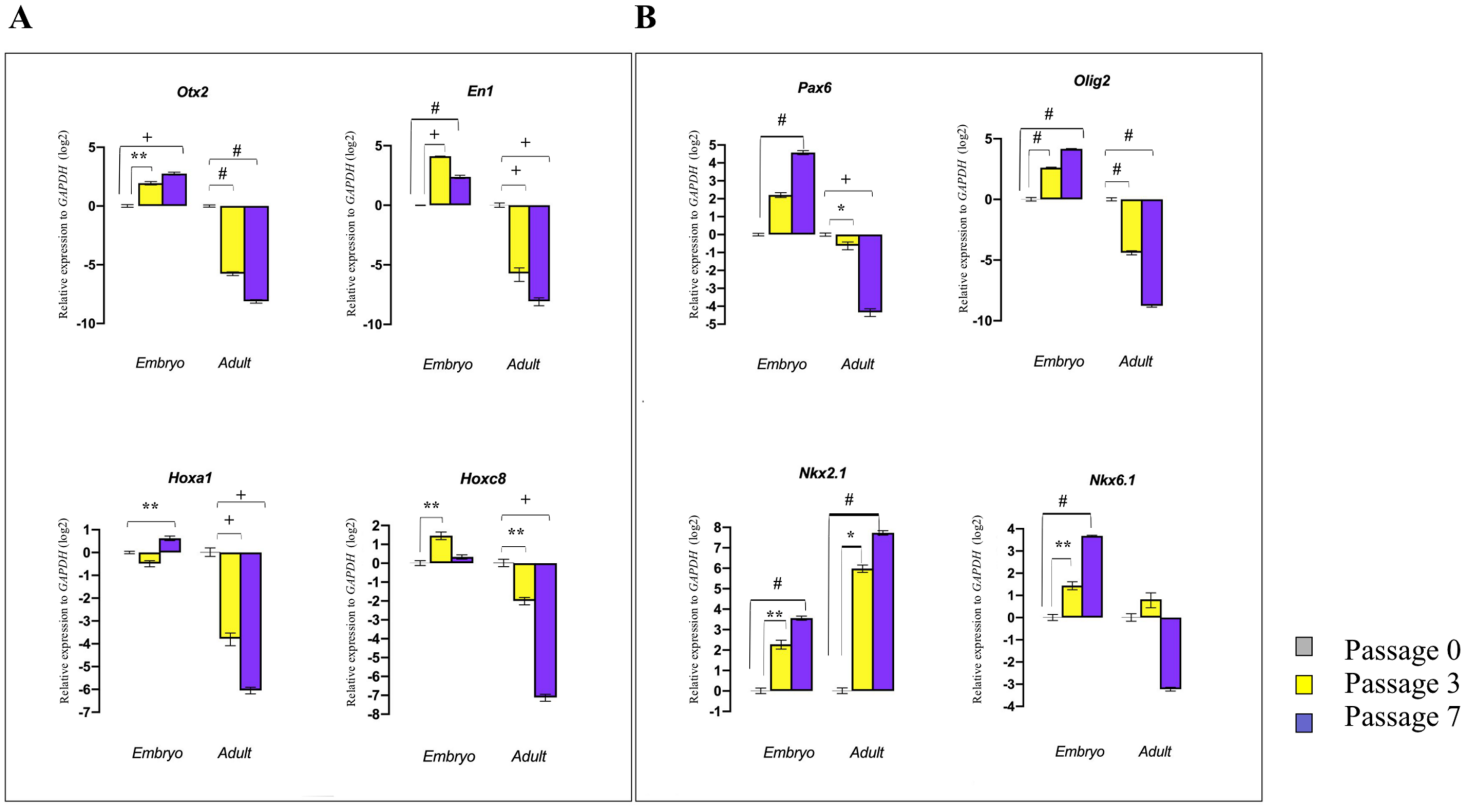
Effect of cell passaging on initial neural patterning of embryonic and adult NSPCs. RT-qPCR analysis of gene expression levels along the RC axis (*Otx2, En1, Hoxa1*, and *Hoxc8*) **(A)** and DV axis (*Pax6, Olig2, Nkx2.1,* and *Nkx6.1*) **(B)** in embryonic and adult-derived NSPCs across different passages indicated that embryonic NSPCs not only retain but also upregulate neural patterning genes at higher passages. In contrast, results for the adult NSPCs displayed downregulation of patterning gene expression with an increase in passage. Data are presented as means ± SEM, normalized to the expression level of *GAPDH* as an internal control, and compared with the expression level of embryonic and adult NSPCs in passage 0. ^*^p < 0.05, ^**^p < 0.01, ^+^*p* < 0.001, and ^#^*p* < 0.0001.

To determine whether dissociated E14 GE-derived NSPCs and adult SVZ-derived NSPCs can maintain their tissue-specific patterning over an extended culturing period, the expression levels of the RC/DV axes-related TFs were evaluated during different passages (P0, P3, and P7) *in vitro.* The results demonstrated that E14 GE-derived NSPCs at higher cell passages exhibited increased expression of patterning genes. In contrast to embryonic NSPCs, SVZ-isolated NSPCs could not retain their neural patterning potential over time (with the exception of *Nkx2.1*, which was highly expressed at higher passages), and the cells at the higher passages lost this property, suggesting once again the adult brain fails to maintain its initial patterning potential over time (Figures 7A,B; Supplementary Table 6).

### 3.10 Potential of neural patterning in suspended vs. attached NSPCs

**Figure 8:**
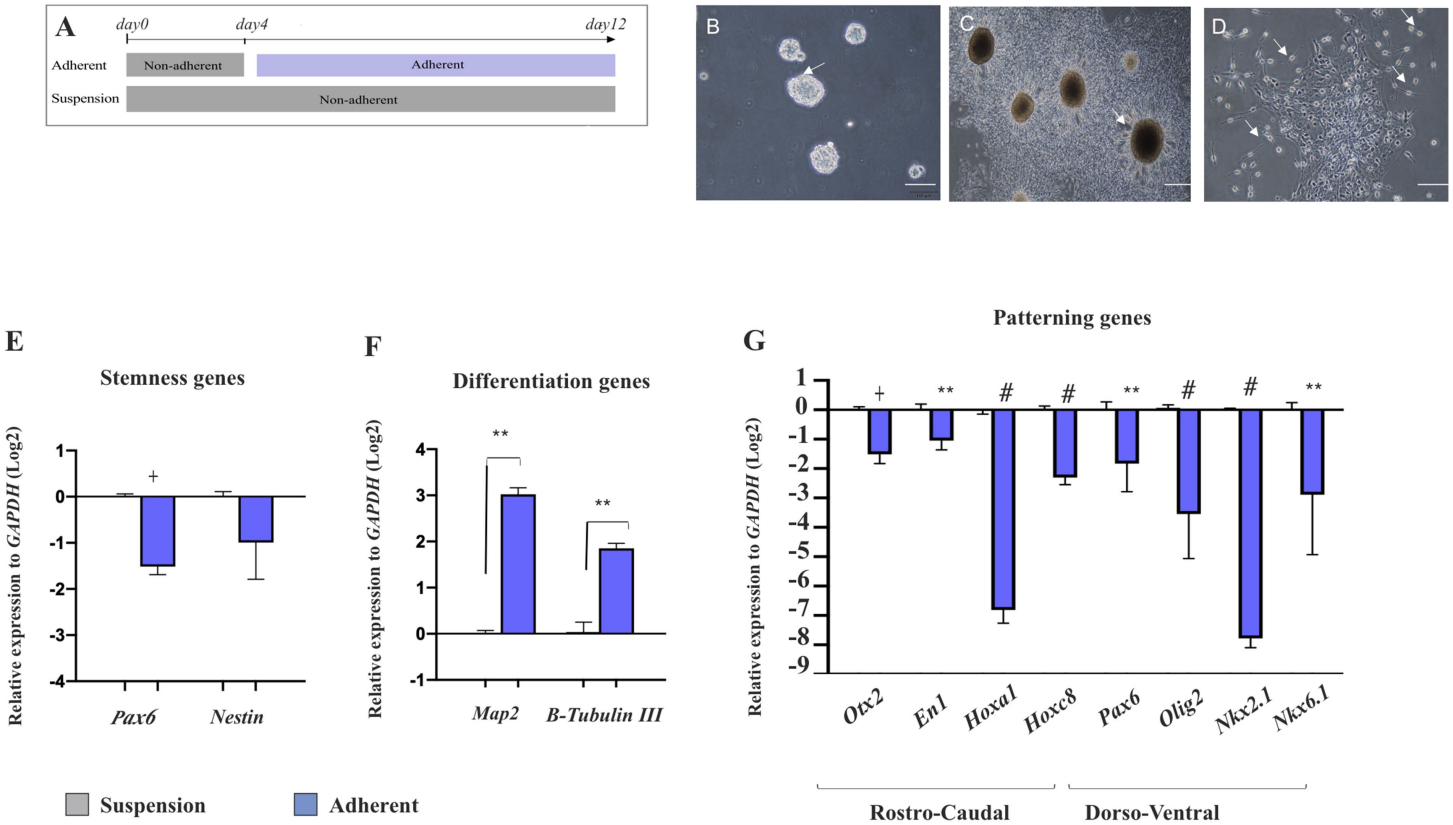
Effect of cell attachment on initial neural patterning of NSPC. Schematic image of the experimental procedure used for NSPC culture in suspension and adherent condition, **(A)**. Phase-contrast images of NSPC cultures after 4 days in both suspension **(B)** and adherent conditions indicate neural-like cells with bright soma and tiny cell processes at the neurosphere margin after 8 days **(C,D)**. RT-qPCR analysis of stemness (*Pax6* and *Nestin*) **(E)** and neuronal differentiation genes (*Map2* and *B-Tubulin III*) **(F)** in E18-derived NSPCs in both suspension and adherent conditions shows that NSPCs tend to alter their stemness to a differentiate state upon attachment. RT-qPCR analysis of gene expression levels along the RC axis (*Otx2, En1, Hoxa1*, and *Hoxc8*) and DV axis (*Pax6, Olig2, Nkx2.1,* and *Nkx6.1*) in embryonic E18- and adult-derived NSPCs in suspension and adherent conditions **(G)** demonstrates that NSPCs lose their preliminary patterning after attachment. Data are presented as means ± SEM, normalized to the expression level of *GAPDH* as an internal control and compared with the expression level of suspended NSCs. ^*^p < 0.05, ^**^p < 0.01, ^+^*p* < 0.001, and ^#^*p* < 0.0001.

To investigate whether NSPCs retain their regional identity after attachment and subsequent differentiation, E14 GE-derived neurospheres in the control group (suspension group) were cultured as suspension for 12 days in Neurobasal medium containing DMEM/F12 complemented with 2% B27, 1% GlutaMAX, 1% NEAA, heparin (1 μL.ml^−1^ of 0.2%,), 100 U.ml^−1^ penicillin, 100 μg.ml^−1^ streptomycin, EGF (20 ng.ml^−1^). In the attachment group, E14 GE-derived neurospheres were cultured in the same medium for 4 days and then transferred onto prewashed 0.1% (w/v) gelatin-coated plates. They were then cultured for an additional 8 days in the same medium supplemented with 5% FBS for attachment (Figure 8A). As shown in Figures 8B,D, compared to the suspended neurospheres, the cells began to extrude from the attached neurospheres and differentiated into neural-like cells by day 12. These cells acquired a neural phenotype with bright soma and short bipolar processes. They lost their neural stemness markers (*Nestin* and *Pax6*) and began to express *B-Tubulin III* and *Map2,* indicating their fate to become neurons (Figures 8E,F). Interestingly, these differentiating neural-like cells were unable to maintain their regional identity and RC/DV patterning TFs, which were initially present in suspended neurosphere–derived NSPCs. This suggests that differentiating NSPCs lose their original region-specific patterning after attachment and differentiation into neural-like cells (Figure 8G; Supplementary Table 7).

## 4 Discussion

In this study, we concentrated on isolating tissue from the GE at E14 and E18, along with the SVZ from both the neonate and adult telencephalon. This approach enabled us to present a temporal profile of neural patterning and examine the changes occurring in developing NSPCs over time. Previously, we found that by using the neurosphere assay, the differentiated cells are unable to proliferate and survive, leading to their decline, while a small number of stem cells do proliferate, forming small clusters that detach from the substrate and create neurosphere (Golmohammadi et al., 2008). We set up experiments using varying numbers of NSPCs to determine the optimal size of neurospheres in all groups. We discovered that using fewer than 200,000 cells/ml during the embryonic stage, or a higher initial cell density during the generation of neonatal and adult neurospheres, resulted in cell death or neurosphere aggregation, respectively (Coles-Takabe et al., 2008). This suggests that neurosphere size is more crucial than initial cell density in producing the desired neurospheres for assay. In particular, it has been reported if the neurospheres become too large, they develop dark centers due to cell death, while the surfaces of healthy neurospheres exhibit micro spikes (Azari et al., 2011b, 2010; Soares et al., 2020). We also obtained similar results and selected both embryonic and adult neurospheres with an average size of 150 μm, represented with a bright margin for further analysis.

To confirm the stemness of NSPCs from the embryonic and adult tissue, we utilized the expression of the *Nestin* gene by the immunocytochemistry assay as highlighted in Park’s report that indicated *Nestin* is fundamental for NSCs survival and self-renewal (Kim et al., 2006; Morshead et al., 1994). However, some reports emphasize that *Nestin* expression may not be reliable as a single marker for all NSCs (Imayoshi et al., 2011). By comparing embryonic and adult neurospheres, we found that the number of embryonic neurospheres was more than two times as high, and this increase is due to a higher proliferation rate of embryonic neurospheres, as we have shown. This discrepancy may also be attributed to BMP signaling, which maintains embryonic NSCs in a proliferative state while promoting adult NSCs in a quiescent state to prevent the exhaustion of stem cells in the brain (Urbán and Guillemot, 2014). In addition, the high expression of FGFR4 in neural stem/precursor cells in the VZ of the dorsal telencephalon is also necessary for self-renewing symmetric division (Lathia et al., 2007).

These results are consistent with previous studies that have reported on the restricted self-renewal capacity and limited lineage potential of adult neurospheres compared to embryonic neurospheres (Tropepe et al., 2000). Kim and Morshead (2003) also indicated that fewer NSCs are achieved from the adult neurospheres. As only a small percentage of cells populate adult neurospheres, this is attributed to NSCs (Kim and Morshead, 2003). In addition, we demonstrated that both embryonic and adult-derived NSPCs display the capacity for differentiation into tri-lineages through protein expression of *B-Tubulin III* for neurons, *GFAP* for astrocytes, and *MBP* for oligodendrocytes. This differentiation potency as functional identities serves as the key hallmark for neurosphere recognition (Rushing and Ihrie, 2016). Taken together, this dual verification of NSCs (proliferation and differentiation) provides them as a suitable source for neural.

Along the rostrocaudal (RC) axis of the developing brain, *Otx2* plays essential roles in the early development of the anterior part of the CNS to being patterned and specified to telencephalon and diencephalon. Additionally, *Hox* genes are involved in the posterior region of the brain, with a function in segmental patterning and subsequent neuronal differentiation at distinct positions along neural tubes (Gavalas et al., 2003). Along the dorsoventral axis (DV), *Pax6* is also key to organizing dorsal identities in the developing brain (Götz et al., 2016). Initially, its expression is all over the neuroepithelium, but finally restricted to the presumptive dorsal axis of the telencephalon and the border between *Pax6* and *Nkx2.1* expression determines the presumptive dorsal and ventral area (Corbin et al., 2003).

In the following experiments, we evaluated the levels of candidate gene expression along the RC (*Otx2*, *En1*, *Hoxa1*, and *Hoxc8*) and DV (*Pax6, Olig2, Nkx2.1*, and *Nkx6.1*) axes in NSPCs and tissues from E14, E18, neonate, and adult stages. The tissue derived from GE and SVZ at different times expressed the rostral (*Otx2*) and the ventral genes (*Olig2* and *NKx2.1*) at high levels. Meanwhile, NSPCs derived from those tissues at appropriate times also expressed high levels of the same transcription factors with minor variation, suggesting that NSPCs can serve as a promising *in vitro* model for neural patterning analysis of the telencephalon. These results align with Azari and Reynolds (2016), indicating that NSPCs can replicate the processes occurring during in vivo neurogenesis and can function as a valuable *in vitro* model (Azari and Reynolds, 2016).

Neural stem cells are temporally patterned in response to extracellular signaling (Ray et al., 2022). Therefore, we posed the question of whether changes in brain structure and function over time would impact its regional identity and whether aging restricts the initial patterning of the prosencephalon and NSCs. The gene expression analysis of the adult brain or brain-derived GE and SVZ for the candidate regional genes indicates a significant downregulation of patterning genes expression, such as *En1*, *Pax6*, *NKX2.1*, and *Olig2*, in the adult brain/SVZ compared to the embryonic brain/GE, suggesting that the adult brain and its SVZ region cannot maintain the initial neural patterning capacity and this potential alters over time. As aging reduces neurogenesis in adult SVZ and hippocampal niches (Bailey et al., 2004; Ben Abdallah et al., 2010; Blackmore and Rietze, 2013; Bondolfi et al., 2004), this decline can be due to enhanced NSC dormancy, reduced NSC self-renewal and survival of their progeny, limited neuronal fate commitment, and finally, NSC perdition (Obernier and Alvarez-Buylla, 2019; Navarro Negredo et al., 2020). The adult neurogenesis process is modulated by multiple physiological stimuli, and the age-related microenvironment significantly affects inherited neural patterning (Urbán and Guillemot, 2014). Studies have shown similarities and differences between embryonic and adult NSC niches and molecular pathways. FGF-2 plays a crucial role in maintaining an undifferentiated state and promoting proliferation. In addition, Shh has a significant effect on cell proliferation, making this signaling pathway pivotal in NSC development (Lathia et al., 2007). Moreover, a recent report indicates that Protein S1, through downregulation of Notch signaling, promotes the balancing of NSC proliferation and differentiation (Zelentsova et al., 2017). However, how these signals change over time in the adult brain and affect the initial neural patterning is not well understood.

Given that growth factors play an important role in the survival of NSPCs by promoting self-renewal and proliferation *in vivo* and *in vitro* (Li H. et al., 2022; Mason, 2007; Tropepe et al., 1999), it has been observed that these growth factors, when combined with other molecules, such as noggin and all-trans-retinoic acid (RA), can influence neural patterning (Okada et al., 2008; Zhao et al., 2019). In our experiment, we aimed to minimize the impact of growth factors on neural patterning by using the minimum possible amounts of EGF/FGF to produce embryonic, neonate, and adult-derived neurospheres. We found that when NSPCs from the adult SVZ are exposed to less than 10 ng/mL of bFGF, they do not proliferate and form the desired neurosphere. Therefore, only EGF was used to promote neurosphere formation from embryonic GE, and later, as SVZ-derived NSPCs became more reliant on additional factors (Martens et al., 2000), a combination of EGF and bFGF was required to produce high-quality neurospheres. However, we cannot rule out the effect of EGF/FGF, even in low doses on neural patterning evaluation which, as mentioned already, was our experimental limitation in generating neurospheres and requires further analysis. Nevertheless, even by using the combination of EGF/FGF on neonate and adult neurospheres, the potential of neural patterning significantly decreased, compared to embryonic neurosphere, suggesting that this potential changes over time, even in the presence of growth factors in the culture medium.

Encinas et al. (2011) also reported that NSC, upon exiting their quiescent state, rapidly underwent a series of asymmetric divisions to produce dividing progeny destined to become neurons and subsequently mature into astrocytes. This alteration led to changes in the size of the NSC pools and showed a dramatic decrease in their populations over time (Encinas et al., 2011). Data indicated that various important molecular and developmental aspects of NSC regional identity are preserved when cultured out of the brain; therefore, regional identity can be considered as cell-intrinsic and may follow of the origin tissue.

To explore whether NSPCs can retain the initial neural patterning, we compared this property in the embryonic and the adult NSPCs over time. We found a notably high patterning genes expression at embryonic, such as *Otx1*, *EN1*, *Hoxa1* and *Olig2, Nkx2.1,* followed by a decline as development proceeds into adulthood, suggesting that aging may impose restrictions on the inherent patterning potential of NSPCs (Chen and Konstantinides, 2022). These findings are in contrast to as described previously by Qian et al. (1998), who indicated that the neurogenesis temporal pattern is preserved in cultured NSPCs (Qian et al., 1998), and in addition, Delgado et al. (2016) declared that NSCs differentiate during development with an expression of regional TFs (Delgado et al., 2016).

We suppose that this decline may be closely linked to alterations in the differentiation status of NSPCs as they transition from the embryonic to the adult stage. We observed that expression of neural stemness markers, *Nestin* and *Pax6*, progressively decreased in NSPCs with aging. Concomitantly, this decline facilitates the differentiation of NSPCs into mature neural cells expressing *B-Tubulin III* and *Map2* over time. These results are consistent with previous research demonstrating that reduction in *Nestin* expression is integral to the differentiation process, ultimately leading to the complete loss of *Nestin* expression as cells mature (Morshead et al., 1994). Furthermore, the study by Duan et al. (2013) highlights the role of *Pax6*, a crucial neurogenic determinant that regulates both the proliferation and differentiation of NSPCs. Their findings indicate that while *Pax6* expression is maintained into adulthood, there is a notable decrease in its expression intensity, underscoring the dynamic changes that occur in NSPCs as they age (Duan et al., 2013).

To characterize whether the neurospheres preserve their preliminary regional identities during a prolonged cultivation *in vitro*, through passaging of neurospheres, we observed that embryonic NSPCs up-regulate the several candidate TFs up to seven passages. This finding highlights the inherent robustness of embryonic NSPCs in maintaining their pluripotency and developmental potential. In contrast, the adult NSCs exhibited a significantly reduced capacity to retain these properties over time. This declined potential of adult NSPCs suggests that aging alters the biological nature of NSCs over extended cultivation and restricts initial regional identity (Kim et al., 2006; Ramalho-Santos et al., 2002). This is manifested that the enhanced passaging could display more structural variations and genomic instability (Merkle et al., 2017; Närvä et al., 2010).

Stem cell therapy is a widely used and promising strategy for treating neurodegenerative disorders, where neural stem cells are transplanted into the degenerated brain either in neurosphere form or through directed differentiation into specific neuronal subtypes from adult NSCs or embryonic stem cells, such as dopaminergic neurons for the treatment of Parkinson’s disease (Nie et al., 2023). However, based on research in the literature, it is less known whether isolated embryonic neurosphere-derived NSPCs can maintain their patterning during attached cultivation. Therefore, we cultured these neurospheres onto a culture dish with a medium containing FBS and found that they lost their initial neural patterning 8 days after attachment. These results suggest that neurospheres can only maintain their patterning potential in suspension conditions and lose it when attached. Delgado et al. (2016) reported that *Nkx2.1* maintained its expression following monolayer differentiation. This finding contrasts with our results, where we observed that this expression was significantly reduced when the neurosphere was attached. We believe this difference may reflect the type of attached neurosphere concerning its origin and culture conditions for expansion and differentiation as the physical niche and cell–cell communication play a crucial role in cellular behavior and differentiation (Delgado et al., 2016). Moreover, we found that the attached neutrosphere began to differentiate into neural-like cells, expressing *Map2* and *B-Tubulin III* following attachment. One possible explanation for this differentiation may be the presence of FBS in the medium used for neurosphere attachment (Seaberg and Van der Kooy, 2002; Tropepe et al., 2001). Delgado also used the same concentration of FBS as we did, but, as mentioned above, the results obtained were different. Additionally, Wnt ligands and receptors are expressed and play their roles in both the expansion and neurogenic phases. The Wnt/b-catenin pathway promotes proliferation via upregulation of cyclin D1, cyclin D2, and c-Myc expression in other systems, while it appears to promote neuronal differentiation via upregulating Ngn1 and Ngn2 expression in the neocortex (Wen et al., 2009). Taken together, how the serum components modulate cell signaling and promote neural differentiation by affecting neural patterning is a subject that requires further analysis.

Despite this investigation, some key questions remain unanswered. For example, we need to address how we can overcome the aging problem to prevent or at least reduce the decline in the inherited potential of neural patterning in NSPCs through manipulation of the culture system. What are the differences at the cellular and molecular levels in the potential for neural patterning between NSPCs derived from SVZ and hippocampal tissue? Which is a better resource for inducing adult NSPCs to exhibit specific neural patterning, leading to differentiation into specific neuron subtypes? Moreover, finally, a clonal segregation of which stem cell type in adult neurospheres provides better sources for achieving the appropriate positional neural patterning, generating the specific neuron subtypes.

## 5 Conclusion

Our findings support the idea that neurogenesis and regional identity are temporally linked. Through the use of the neurosphere assay, we have demonstrated that the temporal neural patterning of NSPCs decreases from embryonic to adult age *in vitro*. Prolonged culturing of adult NSPCs also results in a significant decline in neural patterning potential. Additionally, after attachment and differentiation of embryonic neurospheres, their inherited neural patterning significantly decreases.

## Data Availability

The datasets presented in this study can be found in online repositories. The names of the repository/repositories and accession number(s) can be found in the article/Supplementary material.
