# Supplementary material for: Aging restricts the initial neural patterning potential of developing neural stem and progenitor cells in the adult brain
Source: Front Aging Neurosci. 2025 Jan 23;16:1498308. doi: 10.3389/fnagi.2024.1498308 (PMC11798963; doi:10.3389/fnagi.2024.1498308)
Supplement: Supplementary file 1 [file Table_1.docx]

Supplementary Material

Aging Restricts the Initial Neural Patterning Potential of Developing Neural Stem and Progenitor Cells in the Adult Brain

Saeideh Aran^1^, Mohammad Ghasem Golmohammadi ^2,^ Mohsen Sagha^2^* and Kamran Ghaedi^3^*

^1^ Department of Plant and Animal Biology, Faculty of Biological Science and Technology, University of Isfahan, Isfahan, Iran.

^2^ Research Laboratory for Embryology and Stem Cells, Department of Anatomical Sciences, School of Medicine, Ardabil University of Medical Sciences, Ardabil, Iran.

^3^ Department of Cell and Molecular Biology and Microbiology, Faculty of Biological Science and Technology, University of Isfahan, Isfahan, Iran.

* Correspondence:

Mohsen Sagha

[m.sagha@arums.ac.ir](mailto:m.sagha@arums.ac.ir)

Kamran Ghaedi

[kamranghaedi@sci.ui.ac.ir](mailto:kamranghaedi@sci.ui.ac.ir)

**Table 1.** Number of neurospheres obtained from the culture of NSPCs derived from the embryonic ganglionic eminence and the Adult subventricular zone.

| Mouse Embryo Adult |
| --- |
| 1 729 319  2 707 338  3 679 267  4 686 281  5 703 288  Total 700.8±17.5 298.6 ± 26.03 |

**Table 2.** The size of neurosphere derived from embryonic and adult brain in different days.

| Day In Vitro | Stage | Size (µm) | Mean(µm) | SD (µm) |
| --- | --- | --- | --- | --- |
| Day 2 | Embryo | 42 60 38 55 65 43 50 47 42 62 | 50.4 | ±9.5 |
|  | Adult | 36 15 21 10 23 25 19 17 31 23 | 21.9 | ±7.5 |
| Day 4 | Embryo | 80 78 103 110 100 80 75 95 90 87 | 89.8 | ±12.5 |
|  | Adult | 66 63 75 50 61 58 69 60 65 63 | 63 | ±6.6 |
| Day 6 | Embryo | 143 138 136 148 154 124 146 156 125 141 | 141.1 | ±10.8 |
|  | Adult | 96 70 100 90 88 112 95 76 95 83 | 90.5 | ±12.09 |
| Day 8 | Embryo | 201 182 158 176 164 192 177 153 198 195 | 179.66 | ±17.09 |
|  | Adult | 152 149 135 141 132 145 133 146 138 144 | 141.5 | ±6.8 |

N=3

**Table 3.** The cDNA synthesis Kit (SinaClon # Cat. No: RT5201).

| Materials | Content |
| --- | --- |
| Template: total RNA | 1ng-2μg |
| Random hexamer | 1 μL |
| dNTP | 1 μL |
| Buffer 5x  RNAase-inhibitor  M-Mulv reverse transcriptase  DEPC-treated water | 4 μL  1 μL  1 μL  Top up to 20 μL |

**Table 4.** RT-qPCR (SinaClon # Cat. No: MM2011).

| Materials | Content |
| --- | --- |
| SYBR Green 2X | 10 µL |
| primer Forward | 0.4 µL |
| primer Reverse | 0.4 µL |
| Free Nuclease water  cDNA’s sample | 8.2 µL  1 µL |
